# Spur-of-the-Moment: An Exacting Nasal Intubation

**DOI:** 10.7759/cureus.23922

**Published:** 2022-04-07

**Authors:** Chang-Woo Jang, Allan R Escher, Bruno Bordoni, Jessica Ibanez, Pilar Suz

**Affiliations:** 1 Medicine, University of South Florida Health, Tampa, USA; 2 Anesthesiology: Pain Medicine, H. Lee Moffitt Cancer Center & Research Institute, Tampa, USA; 3 Physical Medicine and Rehabilitation, The Don Carlo Gnocchi Foundation, Milan, ITA; 4 Anesthesiology, H. Lee Moffitt Cancer Center & Research Institute, Tampa, USA

**Keywords:** nasotracheal intubation complications, nasotracheal intubation, nasal septal deviation, nasal septum, nasal bone spur, tube cuff tears, prolonged intubation, intubation complication, nasal spur, spur

## Abstract

Nasotracheal intubations are an important airway management technique in otolaryngologic surgeries and trauma distorting oropharyngeal structures. For those performing these procedures, nasal deformities are not uncommon. This case report highlights an example of recurrent cuff tears that occurred during nasotracheal intubation of a patient with an unknown nasal bone spur. A careful airway analysis with available imaging studies may predict the potential difficulty with nasotracheal intubation. A successful approach to nasotracheal intubation can then be attempted on the contralateral side if a nasal bone spur is present.

## Introduction

In a 1902 article, "Die pernasale tubage," the German surgeon Franz Kuhn first proposed nasal intubation [1]. Magill popularized this method of intubation, in particular, blind nasal intubation, in the 1920s; however, the nasotracheal approach is viewed by many as inherently more traumatic, patient-leading to increased morbidity and perioperative complications [1].

Nasotracheal intubation has historically been used to secure airway access in intraoral or cervical spine pathologies [1]. It offers unimpeded access to the mouth and oropharynx for instrumentation but is not without risk. Complications from a passage of the tube along the nasal floor and posterior nasopharyngeal wall include epistaxis (most common), nasotracheal cuff tears, and trauma: avulsions, lacerations, infection, and tissue damage [2].

Cuff tears have been reported secondary to nasal jewelry, orthodontic implants, misplaced sutures, and bony deformities following Le Fort fractures [3-5]. When possible, the lower pathway (below the inferior turbinate) is the preferred route for intubation; the upper pathway (below the middle turbinate) is less desirable because of the increased risk of trauma such as cerebrospinal fluid (CSF), rhinorrhea, fracture, and bleeding [6]. In 60 patients, a nasogastric tube (NGT)-guided intubation increased tracheal tube passage using the lower pathway in 66.7% of patients [6].

While the risk of complications can be decreased by NGT-guided intubation, we report a most unusual case of three sequential cuff tears during the performance of nasotracheal intubation in a patient with a nasal bone spur [7].

## Case presentation

A 70-year-old male with no prior history of facial trauma was scheduled for elective mandibulectomy in the setting of T3N0M0 mandibular mucosal melanoma. The full surgery would consist of a composite resection of the right and anterior mandible alveolus, including the mouth and buccal mucosa floor. Additional procedures scheduled were a right neck dissection and submental artery island flap reconstruction, 4 X 4cm.

His past medical history was significant for mucosal melanoma along the right mandibular gingiva, diabetes mellitus (type II), hyperlipidemia, gastroesophageal reflux disease (GERD), and chronic insomnia. The patient denied any medication allergies. His current medications were: metformin 1000mg (twice daily), omeprazole 20mg (twice daily), atorvastatin 40mg (once daily), pioglitazone 45mg (once daily), and trazodone 150mg at bedtime. He related a remote history of tobacco usage (50+ years prior). The patient currently uses medical marijuana as an anxiolytic. He denied sleep apnea and reported no personal or family history of anesthesia issues. 

Preoperatively, his vital signs were within normal limits. The patient's weight was 90 kg, height 178.6cm, and body mass index (BMI) 28.2 kg/m2. The airway examination revealed a mouth opening of 5cm, a Mallampati classification of III, and a thyromental distance of >6cm. His dentition was intact and natural, and his cervical range of motion was normal. Auscultation of the patient's lungs revealed clear breath sounds, bilaterally and normal heart sounds. The patient received an American Society of Anesthesiologists (ASA) physical status classification of II. The otolaryngologist requested a nasotracheal approach for intubation based on the nature of the surgery. The left nare was selected due to its larger aperture and ease of respiration per patient. Preoperative preparation for nasal intubation included 0.2mg glycopyrrolate intravenously (IV) and nasal decongestant oxymetazoline, two sprays on each side. Midazolam 2mg IV was given in the preoperative area. After applying standard ASA monitors, the patient received 100% oxygen via face mask for 3 minutes. A smooth IV induction consisted of lidocaine 100mg, propofol 200mg, and 50mcg of remifentanil. After confirming the ability to ventilate the face mask, rocuronium 50mg IV was given and flushed with 10mL of IV fluid.

On the first attempt, a lubricated 7.5mm/10.2mm outer diameter (OD), 380mm length, nasal preformed cuffed endotracheal tube (SunDexTM Indexing System) was placed atraumatically. Intubation was successfully performed and confirmed with direct laryngoscopy. However, an air leak was detected upon auscultation secondary to a cuff tear. For the second attempt, and to reduce the risk of trauma, a smaller 7.0mm/9.6mm OD, 380mm length, a nasal preformed cuffed endotracheal tube was again placed atraumatically. Reassuringly, intubation was accomplished and confirmed with direct laryngoscopy. During auscultation, an air leak was detected secondary to a cuff tear. In an abundance of caution, and even smaller, 6.5 mm/8.8mm OD, 360mm length, a nasal preformed cuffed endotracheal tube was then passed atraumatically-the third intubation, by all accounts, appeared successful. However, during auscultation, those present marveled at the occurrence, yet again, of a cuff tear.

At this point, the physician anesthesiologist selected the right nare for a nasotracheal intubation attempt. On the fourth attempt, a 7.5mm/10.0mm OD, 380mm length, nasal preformed cuffed endotracheal tube (Teleflex®) was successfully placed and confirmed with video laryngoscopy by the anesthesia care and the surgical team. A balanced general anesthetic with sevoflurane and fentanyl proceeded uneventfully. The operation was completed, and throat packs were removed. Sponge, needle, and instrument counts were verified as correct. After extubation, the patient was recovered without incident and transferred to the surgical floor.

The patient's Dobhoff tube was removed after five days, and the patient was discharged home. At a follow-up visit two weeks later, the patient reported continued improvement and was scheduled for adjuvant radiation therapy outpatient.

## Discussion

**Figure 1:**
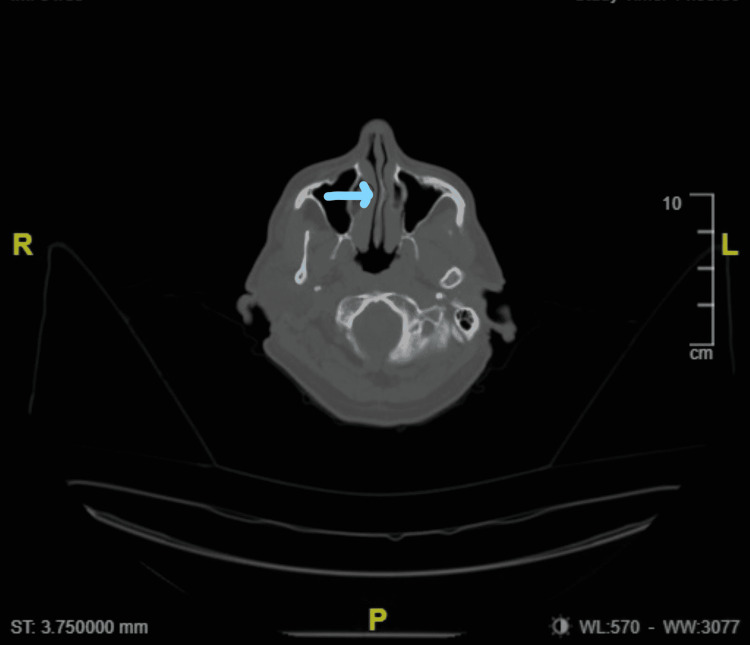
Computerized tomography (CT) of the head Coronal non-contrast head CT image showing left-sided nasal bone spur (blue arrow).

The most frequent anatomical variation of the nasal septum is a deviation of the septal cartilage (80% in adults), which could potentially change facial morphology during growth [8]. The presence of the deviation of the nasal septum can cause various problems, such as breathing difficulties, sinusitis, infections, and turbinate hypertrophy [8]. Another less frequent cartilage anomaly of the septum is the presence of a spur (of a bony nature if posterior, cartilaginous if anterior or mixed bone and cartilage), mostly found on the left side (19.2%) [9]. The finding of the spur deriving from the nasal septum is around 1.2% [10]. The nasal spur can cause pain, particularly rhinogenic contact point ear pain; the latter is an otalgic pain with tinnitus caused by contact between the spur and the intranasal mucosal [11]. Tendentially, in the presence of a bone spur, an endoscopic operation can be carried out. After successful nasotracheal intubation, computerized tomography (CT) of the head revealed a pronounced left-sided nasal bone spur (Figure 1).

The nasotracheal approach to intubation is never without risk or minor trauma. One of the most recurrent events is epistaxis, which is most noticeable if the procedure starts from the left nostril; the right nostril is less prone to epistaxis and demonstrates a faster access rate [11]. Intubation from the right nostril better respects the anatomy of the retropharyngeal wall when the tube is inserted [12]. One of the rarest events is retropharyngeal dissection, which can arise from an anatomical variation of the patient [13].

Tracheal cuff tears can cause a myriad of issues, ranging from aspiration to the inability to deliver adequate tidal volumes, which may necessitate the replacement of the tube. Given this, nasotracheal intubations, when possible, should be preceded by a review of available imaging to map routes that bypass potentially complex nasal deformities and proceed with contingency plans to deal with cuff tears. Preoperative nasal endoscopy may be warranted in select cases with a history of difficulty. In a setting of limited resources, a bedside examination with a medical light may assist the clinician in examining the nasal passages. Some authors have argued for a higher incidence of this issue. Nasal anatomic variations such as bone spurs are not uncommon among the general population, reaching 29.2% in a recent study [8].

## Conclusions

Nasal intubation is vital to secure the airway, particularly in patients presenting for head and neck surgeries. It is also commonly requested for maxillofacial procedures and dental surgeries. Cuff tears are an infrequent and frustrating occurrence during nasal intubation. Repeated difficulties with nasal intubation should prompt a careful analysis of the airway to determine the presence of a nasal bone spur. If available, imaging studies can be a useful tool to guide airway efforts-a successful approach to nasotracheal intubation can then be attempted on the contralateral side.
